# 3-(9*H*-Carbazol-9-yl)-2*H*-chromen-2-one

**DOI:** 10.1107/S1600536811034660

**Published:** 2011-08-27

**Authors:** Julien Letessier, Dieter Schollmeyer, Heiner Detert

**Affiliations:** a University Mainz, Duesbergweg 10-14, 55099 Mainz, Germany

## Abstract

The title compound, C_21_H_13_NO_2_, was prepared as an example of a new synthesis of carbazoles from a cyclic dibenzo-iodo­lium salt *via* a twofold Pd-catalysed aryl­ation of a primary amine. The two essentially planar π-subsystems [maximum deviations from the mean square plane of 0.038 (2) Å in the carbazole and 0.059 (2) Å in the coumarine unit] open a dihedral angle of 63.05 (4)°. Two mol­ecules form a centrosymmetrical pair connected *via* π–π inter­actions between the pyrrole and pyrone rings [centroid–centroid distance = 3.882 (1) Å] and one benzene of the carbazole and the pyrone unit [centroid–centroid distance 3.824 (1) Å]. The lattice is stabilized by C—H⋯O bridging to both coumarin O atoms.

## Related literature

For alkaloids based on the carbazole core, see: Kapil (1971 ▶). For information on carbazoles used as electron-rich and rigid units in functional materials for photoconducting, sensing and luminescence purposes, see: Wakim *et al.* (2004 ▶); Schmitt *et al.* (2008 ▶). For carbazoles and δ-carbolines using the iodo­lium salt route, see Letessier (2011 ▶); Letessier *et al.* (2011 ▶). For the construction of carbazoles and their heteroanalogous derivatives, see: Nissen & Detert (2011 ▶); Dassonneville *et al.* (2011 ▶); Letessier *et al.* (2011 ▶). For the synthesis of annulated heterocycles, see: Nemkovich *et al.* (2009 ▶); Preis *et al.* (2011 ▶).
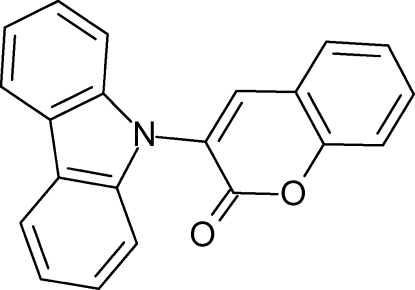


## Experimental

### 

#### Crystal data

C_21_H_13_NO_2_
*M* *_r_* = 311.32
Monoclinic, 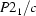

*a* = 8.9451 (12) Å
*b* = 11.5412 (7) Å
*c* = 15.0477 (17) Å
β = 105.871 (12)°
*V* = 1494.3 (3) Å^3^
*Z* = 4
Cu *K*α radiation
μ = 0.72 mm^−1^
*T* = 193 K
0.50 × 0.20 × 0.10 mm

#### Data collection

Enraf–Nonius CAD-4 diffractometer
Absorption correction: ψ scan (*CORINC*; Dräger & Gattow, 1971 ▶) *T*_min_ = 0.716, *T*_max_ = 0.932
2826 measured reflections
2826 independent reflections
2171 reflections with *I* > 2σ(*I*)
3 standard reflections every 60 min  intensity decay: 5%

#### Refinement

*R*[*F* ^2^ > 2σ(*F* ^2^)] = 0.048
*wR*(*F* ^2^) = 0.142
*S* = 1.04
2826 reflections
217 parameters
H-atom parameters constrained
Δρ_max_ = 0.21 e Å^−3^
Δρ_min_ = −0.30 e Å^−3^

### 

Data collection: *CAD-4 Software* (Enraf–Nonius, 1989 ▶); cell refinement: *CAD-4 Software*; data reduction: *CORINC* (Dräger & Gattow, 1971 ▶); program(s) used to solve structure: *SIR97* (Altomare *et al.*, 1999 ▶); program(s) used to refine structure: *SHELXL97* (Sheldrick, 2008 ▶); molecular graphics: *PLATON* (Spek, 2009 ▶); software used to prepare material for publication: *PLATON*.

## Supplementary Material

Crystal structure: contains datablock(s) I, global. DOI: 10.1107/S1600536811034660/bt5627sup1.cif

Structure factors: contains datablock(s) I. DOI: 10.1107/S1600536811034660/bt5627Isup2.hkl

Supplementary material file. DOI: 10.1107/S1600536811034660/bt5627Isup3.cml

Additional supplementary materials:  crystallographic information; 3D view; checkCIF report

## Figures and Tables

**Table 1:** Hydrogen-bond geometry (Å, °)

*D*—H⋯*A*	*D*—H	H⋯*A*	*D*⋯*A*	*D*—H⋯*A*
C15—H15⋯O24^i^	0.95	2.43	3.366 (2)	169
C11—H11⋯O22^ii^	0.95	2.58	3.522 (2)	170

Symmetry codes: (i) 
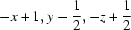
; (ii) 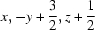.

**Fig. 1.**
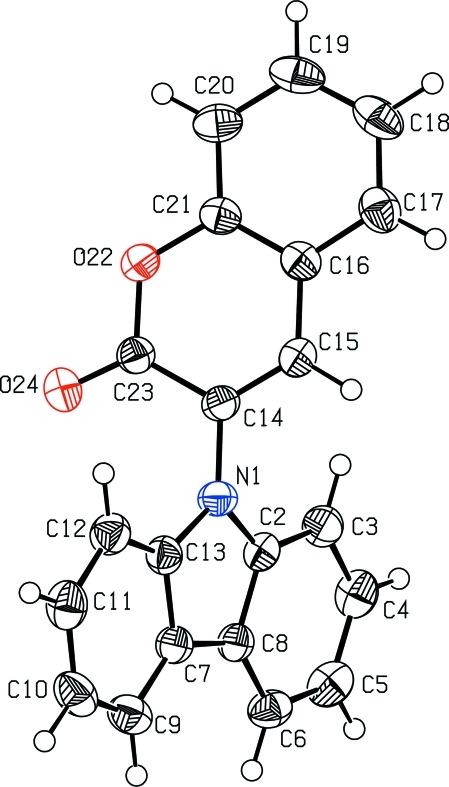
View of compound I. Displacement ellipsoids are drawn at the 50% probability level.

## supplementary crystallographic information

### Comment

The title compound was prepared as a part of a project focused on the synthesis of annulated heterocycles, see: Nemkovich *et al.* (2009); Preis *et al.* (2011). The Pd-catalyzed transformation of dibenzoiodolium salts to carbazoles constitutes a new access to carbazoles, especially 9-substituted carbazoles see: Letessier (2011). This method complements our toolbox for the construction of carbazoles and its heteroanalogous derivatives, see Nissen & Detert (2011); Dassonneville *et al.* (2011) and Letessier *et al.* (2011). Carbazole and coumarine units are essentially planar with maximum deviations from the mean square plane of 0.04Å in the carbazole and 0.06 Å in the coumarine unit. The dihedral angle between the mean planes is 63.1°. Two molecules form a centrosymmetric pair, they are connected *via*π-π interactions between carbazole and coumarin as indicated by the short distances of the centroids of pyrrole and pyrone of 3.88Å and of one benzene of the carbazole and the pyrone of 3.82 Å. The lattice is stabilized by hydrogen bridging to both coumarin oxgen atoms, C11—H11···O22 (2.58 Å) and C15—H15···O24 (2.43 Å).

### Experimental

Dibenzo[b,d]iodol-5-ium trifluoromethanesulfonate (471 mg, 1.10 mmol) (Letessier (2011)), Pd_2_(dba)_3_ (40 mg, 0.044 mmol), Xantphos (76 mg, 0.13 mmol) and Cs_2_CO_3_ (1.07 g, 3.30 mmol) were dissolved in freshly distilled toluene (12 ml) in a sealed tube under argon atmosphere and stirred for 5 min at room temperature. 3-Amino-2*H*-chromen-2-one (213 mg, 1.32 mmol) was added and the mixture was stirred overnight at 373 K. The mixture was cooled to room temperature, filtered trough Celite and concentrated. Purification by silica gel chromatography (petrolether:EtOAc=4:1(v:v)) afforded pure 3-(9*H*-carbazol-9-yl)-2*H*-chromen-2-one as a white crystalline solid (51 mg, 0.17 mmol, 14%).

### Refinement

Hydrogen atoms  were placed at calculated positions with C—H = 0.95 Å (aromatic) or 0.98–0.99 Å (*sp*^3^ C-atom). All H atoms were refined in the riding-model approximation with isotropic displacement parameters (set at 1.2–1.5 times of the *U*_eq_ of the parent atom).

### Crystal data

**Table:**

C_21_H_13_NO_2_	*F*(000) = 648
*M**_r_* = 311.32	*D*_x_ = 1.384 Mg m^−^^3^
Monoclinic, *P*2_1_/*c*	Cu *K*α radiation, λ = 1.54178 Å
Hall symbol: -P 2ybc	Cell parameters from 25 reflections
*a* = 8.9451 (12) Å	θ = 45–50°
*b* = 11.5412 (7) Å	µ = 0.72 mm^−^^1^
*c* = 15.0477 (17) Å	*T* = 193 K
β = 105.871 (12)°	Block, colourless
*V* = 1494.3 (3) Å^3^	0.50 × 0.20 × 0.10 mm
*Z* = 4	

### Data collection

**Table:**

Enraf–Nonius CAD-4 diffractometer	2171 reflections with *I* > 2σ(*I*)
Radiation source: rotating anode	*R*_int_ = 0.000
graphite	θ_max_ = 69.9°, θ_min_ = 4.9°
ω/2θ scans	*h* = 0→10
Absorption correction: ψ scan (CORINC; Dräger & Gattow, 1971)	*k* = 0→14
*T*_min_ = 0.716, *T*_max_ = 0.932	*l* = −18→17
2826 measured reflections	3 standard reflections every 60 min
2826 independent reflections	intensity decay: 5%

### Refinement

**Table:**

Refinement on *F*^2^	Primary atom site location: structure-invariant direct methods
Least-squares matrix: full	Secondary atom site location: difference Fourier map
*R*[*F*^2^ > 2σ(*F*^2^)] = 0.048	Hydrogen site location: inferred from neighbouring sites
*wR*(*F*^2^) = 0.142	H-atom parameters constrained
*S* = 1.04	*w* = 1/[σ^2^(*F*_o_^2^) + (0.0895*P*)^2^ + 0.1404*P*] where *P* = (*F*_o_^2^ + 2*F*_c_^2^)/3
2826 reflections	(Δ/σ)_max_ < 0.001
217 parameters	Δρ_max_ = 0.21 e Å^−^^3^
0 restraints	Δρ_min_ = −0.30 e Å^−^^3^

### Special details

**Table:**

Experimental. ^1^H-NMR (400 MHz, CDCl_3_): *δ* = 8.12 (d, *J*=8.3Hz, 2H), 8.04 (s, 1H), 7.66 (m, 1H), 7.60 (dd, *J*=7.8Hz, *J*=1.5Hz, 1H), 7.51 (d, *J*=8.3Hz, 1H), 7.43 (m, 3H), 7.31 (m, 4H).
Geometry. All e.s.d.'s (except the e.s.d. in the dihedral angle between two l.s. planes) are estimated using the full covariance matrix. The cell e.s.d.'s are taken into account individually in the estimation of e.s.d.'s in distances, angles and torsion angles; correlations between e.s.d.'s in cell parameters are only used when they are defined by crystal symmetry. An approximate (isotropic) treatment of cell e.s.d.'s is used for estimating e.s.d.'s involving l.s. planes.
Refinement. Refinement of *F*^2^ against ALL reflections. The weighted *R*-factor *wR* and goodness of fit *S* are based on *F*^2^, conventional *R*-factors *R* are based on *F*, with *F* set to zero for negative *F*^2^. The threshold expression of *F*^2^ > σ(*F*^2^) is used only for calculating *R*-factors(gt) *etc*. and is not relevant to the choice of reflections for refinement. *R*-factors based on *F*^2^ are statistically about twice as large as those based on *F*, and *R*- factors based on ALL data will be even larger.

### Fractional atomic coordinates and isotropic or equivalent isotropic displacement parameters (Å^2^)

**Table:**

	*x*	*y*	*z*	*U*_iso_*/*U*_eq_	
N1	0.35522 (19)	0.44252 (14)	0.28217 (10)	0.0344 (4)	
C2	0.2569 (2)	0.34605 (16)	0.26142 (12)	0.0326 (4)	
C3	0.2417 (2)	0.26376 (18)	0.19169 (13)	0.0389 (5)	
H3	0.3027	0.2677	0.1491	0.047*	
C4	0.1339 (2)	0.17589 (19)	0.18704 (14)	0.0434 (5)	
H4	0.1218	0.1181	0.1407	0.052*	
C5	0.0427 (2)	0.17044 (19)	0.24880 (14)	0.0431 (5)	
H5	−0.0308	0.1096	0.2435	0.052*	
C6	0.0580 (2)	0.25207 (18)	0.31729 (13)	0.0384 (5)	
H6	−0.0048	0.2484	0.3589	0.046*	
C7	0.1674 (2)	0.34055 (16)	0.32474 (12)	0.0314 (4)	
C8	0.2154 (2)	0.43659 (17)	0.38781 (12)	0.0324 (4)	
C9	0.1733 (2)	0.47311 (19)	0.46582 (13)	0.0410 (5)	
H9	0.0967	0.4321	0.4862	0.049*	
C10	0.2444 (2)	0.5697 (2)	0.51302 (14)	0.0454 (5)	
H10	0.2155	0.5958	0.5659	0.054*	
C11	0.3578 (3)	0.62901 (19)	0.48398 (14)	0.0439 (5)	
H11	0.4059	0.6947	0.5180	0.053*	
C12	0.4028 (2)	0.59507 (17)	0.40682 (14)	0.0394 (5)	
H12	0.4800	0.6363	0.3872	0.047*	
C13	0.3298 (2)	0.49793 (16)	0.35928 (12)	0.0328 (4)	
C14	0.4510 (2)	0.48169 (16)	0.22789 (12)	0.0319 (4)	
C15	0.5679 (2)	0.41781 (16)	0.21408 (13)	0.0338 (4)	
H15	0.5963	0.3477	0.2476	0.041*	
C16	0.6506 (2)	0.45360 (17)	0.14968 (12)	0.0335 (4)	
C17	0.7664 (2)	0.38761 (19)	0.12769 (15)	0.0421 (5)	
H17	0.7984	0.3165	0.1588	0.051*	
C18	0.8348 (2)	0.4251 (2)	0.06098 (16)	0.0497 (6)	
H18	0.9123	0.3793	0.0456	0.060*	
C19	0.7902 (2)	0.5300 (2)	0.01643 (15)	0.0477 (6)	
H19	0.8376	0.5552	−0.0295	0.057*	
C20	0.6781 (2)	0.5980 (2)	0.03778 (14)	0.0408 (5)	
H20	0.6483	0.6699	0.0074	0.049*	
C21	0.6100 (2)	0.55910 (17)	0.10453 (12)	0.0329 (4)	
O22	0.49743 (15)	0.62816 (11)	0.12387 (9)	0.0355 (3)	
C23	0.4108 (2)	0.59401 (16)	0.18148 (12)	0.0329 (4)	
O24	0.30993 (17)	0.65883 (13)	0.19049 (10)	0.0444 (4)	

### Atomic displacement parameters (Å^2^)

**Table:**

	*U*^11^	*U*^22^	*U*^33^	*U*^12^	*U*^13^	*U*^23^
N1	0.0407 (9)	0.0321 (8)	0.0342 (8)	−0.0038 (7)	0.0168 (7)	−0.0014 (6)
C2	0.0344 (9)	0.0291 (9)	0.0332 (9)	0.0008 (7)	0.0075 (7)	0.0032 (7)
C3	0.0452 (11)	0.0376 (11)	0.0344 (10)	0.0005 (9)	0.0116 (8)	−0.0011 (8)
C4	0.0493 (12)	0.0377 (11)	0.0387 (10)	−0.0030 (9)	0.0044 (9)	−0.0039 (8)
C5	0.0384 (11)	0.0419 (12)	0.0434 (11)	−0.0079 (9)	0.0015 (9)	0.0026 (9)
C6	0.0319 (9)	0.0435 (11)	0.0385 (10)	−0.0030 (8)	0.0077 (8)	0.0074 (8)
C7	0.0300 (9)	0.0327 (10)	0.0300 (9)	0.0036 (7)	0.0056 (7)	0.0043 (7)
C8	0.0300 (9)	0.0353 (10)	0.0311 (9)	0.0040 (7)	0.0071 (7)	0.0048 (7)
C9	0.0391 (10)	0.0509 (12)	0.0352 (10)	0.0038 (9)	0.0136 (8)	0.0017 (9)
C10	0.0487 (12)	0.0541 (13)	0.0355 (10)	0.0092 (10)	0.0153 (9)	−0.0059 (9)
C11	0.0516 (12)	0.0377 (11)	0.0390 (11)	0.0013 (10)	0.0067 (9)	−0.0070 (9)
C12	0.0440 (11)	0.0347 (11)	0.0400 (10)	−0.0018 (8)	0.0121 (9)	−0.0008 (8)
C13	0.0350 (9)	0.0319 (10)	0.0312 (9)	0.0035 (8)	0.0089 (7)	0.0010 (7)
C14	0.0360 (9)	0.0301 (10)	0.0311 (9)	−0.0027 (8)	0.0116 (7)	0.0007 (7)
C15	0.0348 (10)	0.0307 (10)	0.0351 (9)	−0.0001 (8)	0.0081 (7)	0.0023 (7)
C16	0.0279 (9)	0.0373 (10)	0.0342 (9)	−0.0026 (8)	0.0065 (7)	−0.0036 (8)
C17	0.0332 (10)	0.0436 (12)	0.0483 (12)	0.0012 (9)	0.0088 (9)	−0.0051 (9)
C18	0.0327 (10)	0.0636 (15)	0.0574 (13)	−0.0040 (10)	0.0199 (10)	−0.0120 (11)
C19	0.0363 (10)	0.0658 (15)	0.0451 (11)	−0.0154 (10)	0.0183 (9)	−0.0081 (10)
C20	0.0367 (10)	0.0480 (12)	0.0375 (10)	−0.0120 (9)	0.0097 (8)	−0.0006 (9)
C21	0.0284 (9)	0.0375 (10)	0.0324 (9)	−0.0051 (8)	0.0074 (7)	−0.0032 (7)
O22	0.0365 (7)	0.0329 (7)	0.0384 (7)	0.0004 (6)	0.0125 (6)	0.0054 (5)
C23	0.0341 (9)	0.0320 (10)	0.0333 (9)	−0.0012 (8)	0.0106 (7)	−0.0002 (7)
O24	0.0466 (8)	0.0391 (8)	0.0512 (8)	0.0094 (6)	0.0197 (7)	0.0023 (6)

### Geometric parameters (Å, °)

**Table:**

N1—C13	1.397 (2)	C11—H11	0.9500
N1—C2	1.400 (2)	C12—C13	1.392 (3)
N1—C14	1.410 (2)	C12—H12	0.9500
C2—C3	1.394 (3)	C14—C15	1.342 (3)
C2—C7	1.404 (3)	C14—C23	1.470 (3)
C3—C4	1.388 (3)	C15—C16	1.431 (3)
C3—H3	0.9500	C15—H15	0.9500
C4—C5	1.395 (3)	C16—C21	1.393 (3)
C4—H4	0.9500	C16—C17	1.397 (3)
C5—C6	1.375 (3)	C17—C18	1.380 (3)
C5—H5	0.9500	C17—H17	0.9500
C6—C7	1.397 (3)	C18—C19	1.388 (3)
C6—H6	0.9500	C18—H18	0.9500
C7—C8	1.446 (3)	C19—C20	1.379 (3)
C8—C9	1.393 (3)	C19—H19	0.9500
C8—C13	1.405 (3)	C20—C21	1.384 (3)
C9—C10	1.380 (3)	C20—H20	0.9500
C9—H9	0.9500	C21—O22	1.376 (2)
C10—C11	1.390 (3)	O22—C23	1.369 (2)
C10—H10	0.9500	C23—O24	1.208 (2)
C11—C12	1.385 (3)		
			
C13—N1—C2	108.33 (15)	C11—C12—H12	121.4
C13—N1—C14	126.66 (16)	C13—C12—H12	121.4
C2—N1—C14	124.73 (15)	C12—C13—N1	129.46 (18)
C3—C2—N1	129.48 (18)	C12—C13—C8	121.80 (17)
C3—C2—C7	121.57 (18)	N1—C13—C8	108.71 (16)
N1—C2—C7	108.95 (16)	C15—C14—N1	122.45 (17)
C4—C3—C2	117.34 (19)	C15—C14—C23	120.71 (16)
C4—C3—H3	121.3	N1—C14—C23	116.74 (16)
C2—C3—H3	121.3	C14—C15—C16	121.02 (17)
C3—C4—C5	121.60 (19)	C14—C15—H15	119.5
C3—C4—H4	119.2	C16—C15—H15	119.5
C5—C4—H4	119.2	C21—C16—C17	118.19 (18)
C6—C5—C4	120.78 (19)	C21—C16—C15	117.93 (17)
C6—C5—H5	119.6	C17—C16—C15	123.86 (19)
C4—C5—H5	119.6	C18—C17—C16	120.4 (2)
C5—C6—C7	118.99 (19)	C18—C17—H17	119.8
C5—C6—H6	120.5	C16—C17—H17	119.8
C7—C6—H6	120.5	C17—C18—C19	119.9 (2)
C6—C7—C2	119.70 (18)	C17—C18—H18	120.0
C6—C7—C8	133.52 (18)	C19—C18—H18	120.0
C2—C7—C8	106.77 (16)	C20—C19—C18	121.03 (19)
C9—C8—C13	119.51 (18)	C20—C19—H19	119.5
C9—C8—C7	133.24 (18)	C18—C19—H19	119.5
C13—C8—C7	107.23 (16)	C19—C20—C21	118.5 (2)
C10—C9—C8	119.06 (19)	C19—C20—H20	120.8
C10—C9—H9	120.5	C21—C20—H20	120.8
C8—C9—H9	120.5	O22—C21—C20	117.27 (18)
C9—C10—C11	120.62 (18)	O22—C21—C16	120.75 (16)
C9—C10—H10	119.7	C20—C21—C16	121.97 (18)
C11—C10—H10	119.7	C23—O22—C21	122.72 (14)
C12—C11—C10	121.9 (2)	O24—C23—O22	117.53 (17)
C12—C11—H11	119.1	O24—C23—C14	125.86 (17)
C10—C11—H11	119.1	O22—C23—C14	116.60 (16)
C11—C12—C13	117.11 (19)		
			
C13—N1—C2—C3	−179.12 (19)	C7—C8—C13—C12	−178.87 (17)
C14—N1—C2—C3	6.6 (3)	C9—C8—C13—N1	177.92 (16)
C13—N1—C2—C7	0.6 (2)	C7—C8—C13—N1	−0.8 (2)
C14—N1—C2—C7	−173.62 (16)	C13—N1—C14—C15	123.2 (2)
N1—C2—C3—C4	179.88 (18)	C2—N1—C14—C15	−63.6 (3)
C7—C2—C3—C4	0.1 (3)	C13—N1—C14—C23	−60.6 (2)
C2—C3—C4—C5	0.7 (3)	C2—N1—C14—C23	112.6 (2)
C3—C4—C5—C6	−0.6 (3)	N1—C14—C15—C16	172.20 (16)
C4—C5—C6—C7	−0.5 (3)	C23—C14—C15—C16	−3.9 (3)
C5—C6—C7—C2	1.3 (3)	C14—C15—C16—C21	2.5 (3)
C5—C6—C7—C8	−178.49 (19)	C14—C15—C16—C17	−175.69 (18)
C3—C2—C7—C6	−1.2 (3)	C21—C16—C17—C18	−1.9 (3)
N1—C2—C7—C6	179.02 (16)	C15—C16—C17—C18	176.35 (18)
C3—C2—C7—C8	178.68 (17)	C16—C17—C18—C19	1.0 (3)
N1—C2—C7—C8	−1.1 (2)	C17—C18—C19—C20	0.1 (3)
C6—C7—C8—C9	2.6 (4)	C18—C19—C20—C21	−0.4 (3)
C2—C7—C8—C9	−177.3 (2)	C19—C20—C21—O22	−179.40 (16)
C6—C7—C8—C13	−179.0 (2)	C19—C20—C21—C16	−0.5 (3)
C2—C7—C8—C13	1.2 (2)	C17—C16—C21—O22	−179.53 (16)
C13—C8—C9—C10	0.5 (3)	C15—C16—C21—O22	2.1 (3)
C7—C8—C9—C10	178.82 (19)	C17—C16—C21—C20	1.6 (3)
C8—C9—C10—C11	−0.8 (3)	C15—C16—C21—C20	−176.71 (17)
C9—C10—C11—C12	0.7 (3)	C20—C21—O22—C23	173.28 (16)
C10—C11—C12—C13	−0.3 (3)	C16—C21—O22—C23	−5.6 (3)
C11—C12—C13—N1	−177.60 (19)	C21—O22—C23—O24	−176.57 (16)
C11—C12—C13—C8	0.1 (3)	C21—O22—C23—C14	4.2 (2)
C2—N1—C13—C12	178.00 (19)	C15—C14—C23—O24	−178.58 (19)
C14—N1—C13—C12	−7.9 (3)	N1—C14—C23—O24	5.1 (3)
C2—N1—C13—C8	0.1 (2)	C15—C14—C23—O22	0.6 (3)
C14—N1—C13—C8	174.22 (16)	N1—C14—C23—O22	−175.71 (15)
C9—C8—C13—C12	−0.2 (3)		

### Hydrogen-bond geometry (Å, °)

**Table:**

*D*—H···*A*	*D*—H	H···*A*	*D*···*A*	*D*—H···*A*
C15—H15···O24^i^	0.95	2.43	3.366 (2)	169
C11—H11···O22^ii^	0.95	2.58	3.522 (2)	170

Symmetry codes: (i) −*x*+1, *y*−1/2, −*z*+1/2; (ii) *x*, −*y*+3/2, *z*+1/2.

## References

[bb1] Altomare, A., Burla, M. C., Camalli, M., Cascarano, G. L., Giacovazzo, C., Guagliardi, A., Moliterni, A. G. G., Polidori, G. & Spagna, R. (1999). *J. Appl. Cryst.* **32**, 115–119.

[bb2] Dassonneville, B., Witulski, B. & Detert, H. (2011). *Eur. J. Org. Chem.* pp. 2836–2844.

[bb3] Dräger, M. & Gattow, G. (1971). *Acta Chem. Scand.* **25**, 761–762.

[bb4] Enraf–Nonius (1989). *CAD-4 Software* Enraf–Nonius, Delft, The Netherlands.

[bb5] Kapil, R. S. (1971). *The Alkaloids*, Vol. 13, pp. 273–302. New York: Academic Press.

[bb6] Letessier, J. (2011). PhD thesis. University of Mainz, Germany.

[bb7] Letessier, J., Schollmeyer, D. & Detert, H. (2011). *Acta Cryst.* E**67**, o2341. 10.1107/S1600536811032107 PMC3200621 22058962

[bb8] Nemkovich, N. A., Kruchenok, Yu. V., Sobchuk, A. N., Detert, H., Wrobel, N. & Chernyavskii, E. A. (2009). *Opt. Spectrosc* **107**, 275–281.

[bb9] Nissen, F. & Detert, H. (2011). *Eur. J. Org. Chem.* pp. 2845–2853.

[bb10] Preis, J., Schollmeyer, D. & Detert, H. (2011). *Acta Cryst.* E**67**, o987. 10.1107/S1600536811010683 PMC3099827 21754244

[bb11] Schmitt, V., Glang, S., Preis, J. & Detert, H. (2008). *Sens. Lett* **6**, 1–7.

[bb12] Sheldrick, G. M. (2008). *Acta Cryst.* A**64**, 112–122. 10.1107/S0108767307043930 18156677

[bb13] Spek, A. L. (2009). *Acta Cryst.* D**65**, 148–155. 10.1107/S090744490804362X PMC2631630 19171970

[bb14] Wakim, S., Bouichard, J., Simard, M., Drolet, N., Tao, Y. & Leclerc, M. (2004). *Chem. Mater.* **16**, 4386–4388.

